# Predictors and tactics for revision surgery in lateral lumbar interbody fusion

**DOI:** 10.1186/s12891-022-06052-8

**Published:** 2022-12-17

**Authors:** Weijian Wang, Jiaqi Li, Yafei Xu, Yun Luo, Wenyuan Ding, Wei Zhang

**Affiliations:** Department of Spinal Surgery, Hebei Medical University Third Affiliated Hospital, 050000 Shijiazhuang, Hebei Province China

**Keywords:** Complication, Lateral lumbar interbody fusion, Revision, Risk factor, Tactic

## Abstract

**Background:**

The purpose of this study is to analyze the factors affecting the revision of lateral lumbar interbody fusion (LLIF), and to summarize the complications and decision-making strategies for revision surgery after LLIF.

**Methods:**

We retrospectively reviewed 21 cases suffered from a revision surgery after LLIF in our department from May 2017 to June 2020, with a mean follow-up of 14 months (12-25months). We collected X-ray plain films, CT (computed tomography), MRI (magnetic resonance imaging) and medical records of all patients undergoing LLIF surgery, then analyzed the reasons for revision and summarized the revision strategies in different situations. We analysed correlations between revision surgery and several factors, including age, body mass index (BMI), sex, bone quality, mode of internal fixation, spinal stenosis, postperative foraminal stenosis, disc height. Then we brought the different indicators into logistic regression to find out the risk factors of revision after LLIF. All these patients were evaluated by Quality-of-life outcomes. Univariate statistical analysis was performed using T-tests, Mann-Whitney U tests and Chi square tests.

**Results:**

Of the 209 cases of LLIF, 21 patients underwent postoperative revision. All revision surgeries were successfully completed. The reasons for revision included vascular injury, unsatistactory implant placement, internal spinal instrumentation failure, cage migration, indirect decompression failure and infection. Indirect decompression failure was the most common indications for revision. Clinical status was apparently improved in ODI scores and VAS scores. Revision surgery did not impact long-term effect and satisfaction. Postoperative foraminal stenosis is a positive predictor for a revision surgical procedure.

**Conclusion:**

Patients with postoperative foraminal stenosis are at higher risk of undergoing revision surgery after lateral lumbar interbody fusion. The correct choice of revision surgery can achieve satisfactory clinical results.

## Background

Lateral lumbar interbody fusion (LLIF) was first described by McAfee in 1998 [1]. Its surgical approach is through the retroperitoneal space to the lateral side of the lumbar spine, including two surgical methods: passing through the anterior psoas major muscle and abdominal great vascular space (oblique lumbar interbody fusion, OLIF) and splitting the psoas major muscle (extreme lateral interbody fusion, XLIF). Compared with the traditional posterior surgery, it has the advantages of less injury, no influence on the stable structure and posterior muscles of the spine, and quick recovery [2]. The incidence of LLIF complications varies greatly, and the most common causes of revision were persistent radiculopathy and symptomatic implant subsidence [3–5]. Preoperative foraminal stenosis and postoperative graft subsidence are considered to be risk factors for LLIF revision [6, 7]. Although the secondary operation from the original surgical approach can reduce the additional injury, due to the small incision and the complex surgical approach, it becomes the difficulty of revision. In previous studies, the proportion of additional posterior laminectomy after LLIF was 0 to 60% [8–11]. In revision surgery, the choice of lateral anterior approach or posterior approach, as well as the addition of internal fixation and other surgical strategies are very important for the effect of revision. At present, some studies described the situation of reoperation after LLIF [5, 6, 12, 13]. In the choice of revision methods, most of them were treated with direct decompression and fusion via posterior approach, but there is a lack of consensus on which technology should be adopted and when. Overall, revision surgery for LLIF patients remains a case-by-case, relying on surgeons and non-standardized procedures.

## Methods

### Study design and study population

This is a retrospective review of prospectively acquired data for consecutive patients undergoing a revision after LLIF. The purpose of this paper is to share the experience of revision due to different complications after LLIF, propose revision strategies according to clinical experience, and analyze the factors affecting reoperation after LLIF. Inclusion criteria: (1) The age is more than 30 years old and less than 80 years old. (2) The patients underwent LLIF because of discogenic back pain, lumbar disc herniation, degenerative lumbar spinal stenosis and degenerative lumbar spondylolisthesis. (3) The patients were followed up for more than 1 year. Exclusion criteria: (1) Prolapse of lumbar intervertebral disc with prolapse of nucleus pulposus. (2) Patients with severe bony stenosis, severe degenerative scoliosis > 40°, high-grade spondylolisthesis(Meyerding grade III or grade IV), severe lumbar osteoporosis. (3) Previous operations on adjacent segments. (4) Incomplete follow-up data. We treated 209 patients with LLIF from May 2017 to June 2020. Among them, 21 patients underwent revision after operation. This study was approved by the Medical Ethics Committee of the Third Hospital of Hebei Medical University (2021-009-1) .

### Description of surgery

In all LLIF operations, cage with allogeneic bone was placed into the intervertebral space through a single incision. The curvature of the cage (CLYDESDALE [MedtronicInc, Minneapolis,Minnesota,USA]) was 6 °and the width was 18 mm. The cage length (45 or 50 mm) was selected according to the distance between the edges of the vertebrae, and the cage height (12 or 14 mm) was selected according to the maximum test height suitable for intervertebral space without excessive force. Different revisions were performed according to different reasons, including adjusting the position of screws or implants, re-direct decompression, secondary internal fixation, focal debridement, irrigation and drainage, etc. All operations were performed by 3 fellowship-trained spinal surgeons with performing LLIF surgery.

### Data collection and analysis

We collected patients’ demographic characteristics, surgical date, mode of internal fixation, complications, reoperation dates, health-related quality-of-life outcomes and radiographic parameters. Revision was defined as an unplanned non-immediate surgery resulting from the primary surgical procedure. Patients’demographic characteristics included sex, age, body mass index (BMI). Radiographic parameters included X-ray plain film, CT and MRI of each patient were collected preoperative, immediately after operation and each follow-up period (3 months, 6 months and 1 year after operation). The patients whose spinal canal area is less than 100mm^2^ in axial MRI are defined as spinal stenosis [14]. According to the sagittal MRI, moderate and severe foraminal stenosis is defined as “perineural fat occlusion without morphological changes in both vertical and transverse directions” or “with rootlets collapse and morphological change” [15]. Quality-of-life outcomes, including the Oswestry Disability Index (ODI), Visual Analogue Scale (VAS)were collected at all time points (baseline, six weeks, six months and a year after operation). The NASS satisfaction questionnaire [16] was used to measure patients’ satisfaction with the results 12 months after operation. Level 1, the operation meets my expectations; Level 2, I haven’t improved as much as I wanted, but I will undergo the same operation to get the same results; Level 3, the operation helps, but I will not undergo the same operation for the same result; Level 4 satisfaction is defined as “my condition is the same or worse than before the operation”.

### Data analysis

According to the clinical and imaging data of the patients before and after the operation, the causes of 21 revision patients were classified. At the same time, the revision methods that should be selected for different complications were summarized, and the strategies of revision surgery were analyzed. Statistical analysis was performed to identify differences among patients who underwent revision surgery following LLIF and those who did not. Differences in categorical data were evaluated using the Chi square tests. The continuous data were tested for their normality first. According to whether the data were normally distributed, independent t-test or nonparametric test was used. The statistical significance level was set at *p* < 0.05. Variables with p values < 0.15 on univariate analysis were considered potential revision predictors and were evaluated during multivariate logistic regression model building. ODI and VAS between the preoperative and postoperative periods in revision group were tested with the repeated measure ANOVA. All statistical analysis was performed in SPSS Statistics for Windows (version 26.0, IBM Corp).

## Results

### Criteria

A total of 209 patients were enrolled in our study: 177 underwent a single-level procedure, 26 underwent a 2-level procedure and 6 underwent a 3-level procedure. Of the 209 patients, 21 patients underwent revision surgery. The average interval between the first and second operations was 43d. Univariate correlation analysis was performed on age, BMI, gender, bone quality, mode of internal fixation, preoperative disc height, foraminal stenosis, spinal stenosis, and revision. Table 1 shows potential revision predictors from univariate analysis that were included in the multivariate regression. The average age of the revision group was 55.19 years old, females accounted for 61.9%, and the average BMI was 26.28; the average age of the unrevised group was 55.66 years old, females accounted for 57.4%, and the average BMI was 26.39. There were more women in both groups, and there was no significant difference in gender between the two groups (*p* = 0.695). There was no significant difference in age, BMI, internal fixation, disc height and bone quality between the two groups. There were differences in the proportion of spinal stenosis and foraminal stenosis (*p* < 0.15) between the two groups. The data of preoperative foraminal stenosis and spinal stenosis were analyzed by multiple logistic regression to determine the risk factors of revision after LLIF.

**Table 1:** Univariate analysis regarding risk factors for revision

Parameter	Number	REVISION		*P*
		YES (*n* = 21)	NO (*n* = 188)	
Gender				0.695^$^
Male	88	8	80	
Female	121	13	108	
Age (year)		55.19 ± 12.83	55.66 ± 11.11	0.582^&^
BMI (kg/m^2^)		26.28 ± 2.14	26.39 ± 2.74	0.389^&^
Smoke	39	5	34	0.523^$^
Disc height(mm)		7.99 ± 1.97	8.25 ± 1.42	0.186^&^
**Moderate and severe foraminal Stenosis(%)**	40	**14 (66.7%)**	**73 (38.8%)**	** < 0.01**^**$**^
**Spinal Stenosis(%)**	71	**13 (61.9%)**	**86 (45.7%)**	**0.120**^**$**^
Internal fixation mode				0.352^$^
Stand Alone	69	10	59	
Lateral plating	31	3	28	
Cage with Fin device	62	6	56	
Posterior internal fixation	47	2	45	
Bone quality				0.392^$^
Normal	130	10	120	
Osteopenia	54	6	48	
Osteoporosis	25	4	21	

^$^Pearson Chi-square test, ^&^Mann–Whitney Test, BMI, body mass index, Boldface indicates statistical significance

### Causes and results for revision

The Table 2 shows six causes for revision. 21 patients underwent revision of LLIF, and different operations were performed according to different reasons, including adjusting the position of screws or implants, re-direct decompression, secondary internal fixation, focal debridement, etc. One patient with lumbar spinal stenosis underwent revision 6 days after operation and had intraoperative complications of peritoneal rupture, which was sutured in time, and no visceral and vascular injury was found. The rest of the revision operations were completed successfully and no intraoperative complications occurred. In the revision group, the average follow-up time was 14 months, all patients were fused, and no post-revisionstatist complications occurred.

**Table 2:** Causes for revision

Cause	Number	Proportion
Vascular injury	2	9.5%
Unsatistactory implant placement	3	14.3%
Internal spinal instrumentation failure	2	9.5%
Cage migration	3	14.3%
Indirect decompression failure	10	47.6%
Infection	1	4.8%

### Logistic regression

The significant variables in single factor analysis were postoperative foraminal stenosis and spinal stenosis, which were included in multivariate analysis. Table 3 shows results of multivariate logistic regression of independent predictors of a revision surgical procedure. The results showed that postoperative foraminal stenosis was an independent risk factor for revision.

**Table 3:** Multivariate analysis for early revision risk factors

Factor	Odds ratio	*p*-value
**Foraminal Stenosis**	**3.906(1.452 to 10.511)**	**0.007**
Spinal Stenosis	2.605(0.987 to 6.879)	0.053

Boldface indicates statistical significance

### Quality-of-life outcomes

**Fig. 1:**
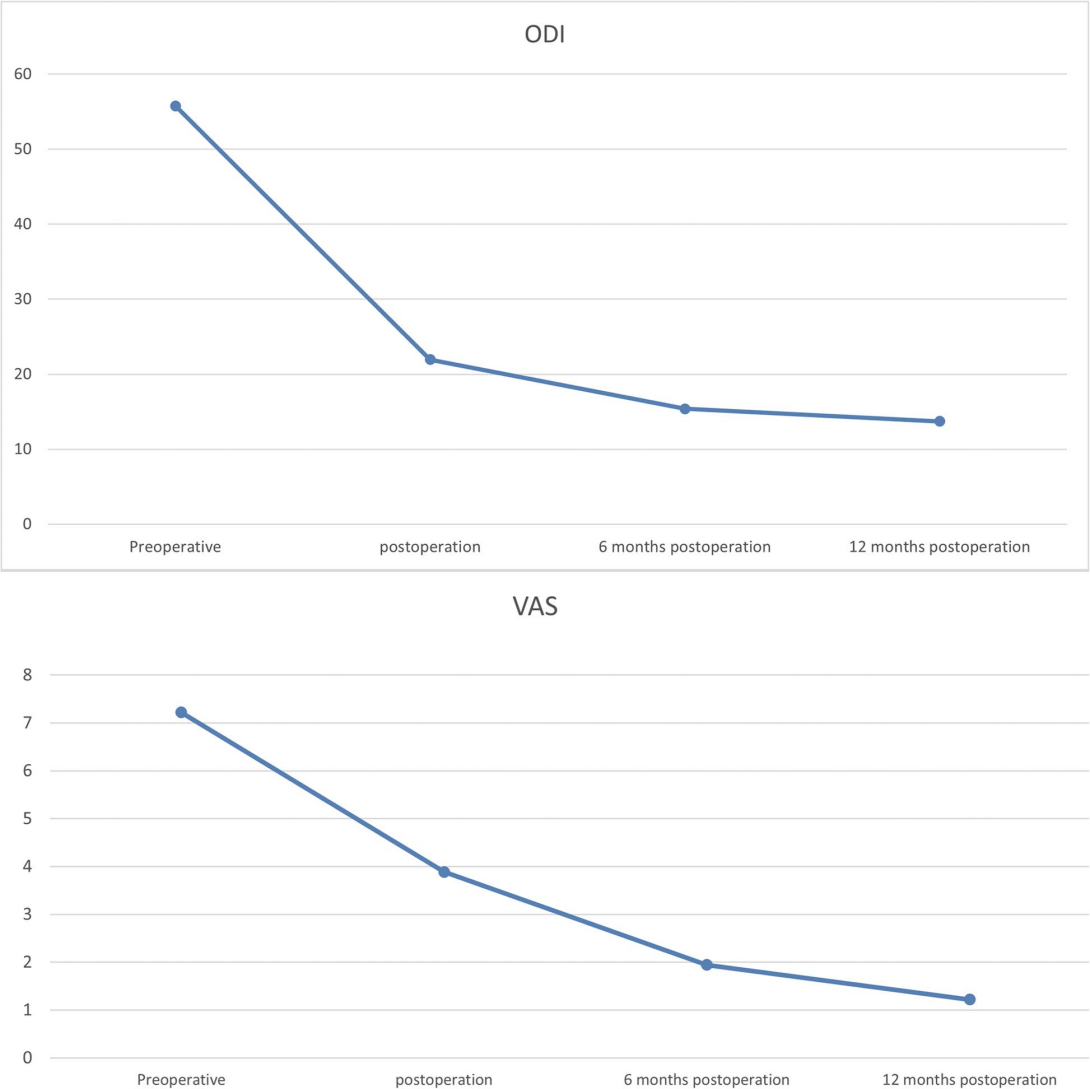
Postoperative ODI and VAS were significantly reduced compared with those of preoperation

Figure 1; Table 4 show clinical outcomes in the revision and nonrevision groups over time. There was no significant difference in preoperative quality-of-life outcomes between the two groups, as measured by the ODI (*p* = 0.532), and VAS (*p* = 0.771). The ODI (*p* < 0.001) and the VAS (*p* < 0.001) during each follow-up period after operation in both groups was significantly reduced compared with that before operation. There was no significant difference in satisfaction between the two groups at 12 months after operation (*p* = 0.869).

**Table 4:** Summary of clinical outcomes

Clinical Outcomes	Preoperative	6 Weeks Postoperatively	6 Months Postoperatively	12 Months Postoperatively	*P*
ODI	54.43 ± 9.68a	21.62 ± 6.18b	15.24 ± 3.33c	13.19 ± 2.40d	** < 0.01**^**#**^
VAS	7.38 ± 1.07a	3.80 ± 1.66b	1.94 ± 0.97c	1.28 ± 0.72c	** < 0.01**^**#**^
NASA	—	—	—	-0.967	**0.334**

*VAS* visual analog scale, *ODI* Oswestry Disability Index

^**#**^repeated measure ANOVA, at least 1 identical subscript letter denoted no significant difference from each other

## Discussion

In our study, 10.05% of patients after LLIF underwent revision surgery, all within one year after the first operation. This is similar to the previous studies reported that the revision rate after LLIF is 3.4–26.0% and the most common complication is transient neurological symptoms [6, 7, 12, 13, 17–19]. The demographic characteristic data were similar between the revision group and the non-revision group, and there was a significant difference in the proportion of preoperative spinal canal stenosis and postoperative foraminal stenosis. In addition, the reasons for revision include vascular injury, unsatistactory implant placement, internal spinal instrumentation displacement, cage migration and infection. The most common cause of revision is indirect decompression failure, accounting for 47.6%. In the long-term follow-up, the revision did not affect the postoperative recovery and satisfaction of patients. We will now discuss our strategies for revision in different complications.

### Indirect decompression failure

Overall reported incidence of indirect decompression failure (IDF) was 9%. The reasons for IDF included endplate collapse, osteoporosis, severe foraminal stenosis, inadequately restored disc and bony lateral recess stenosis [10, 20–23]. LLIF allows the surgeon to place a large cage in the intervertebral space to fully restore the height of the intervertebral space and intervertebral foramen, and at the same time tighten the ligament tissue and release the pressure in the spinal canal, so as to achieve a good effect of indirect decompression [10, 21, 24]. Since LLIF does not directly decompress the lamina, the symptoms may persist or even worsen after IDF. In the choice of revision mode, we need to choose the corresponding method according to the causes of IDF. In order to relieve neurological symptoms, most of the previous studies were supplemented by posterior direct decompression. In addition to the traditional posterior decompression, minimally invasive decompression can also be selected. When the diagnosis is clear, we choose intervertebral foramen endoscope to do intervertebral foramen plasty, and we can also use unlateral biportal endoscoic (UBE) to decompress the lamina. And posterior pedicle screw fixation can prevent further intervertebral space collapse due to osteoporosis and endplate injury after operation.

### Postoperative infection

**Fig. 2:**
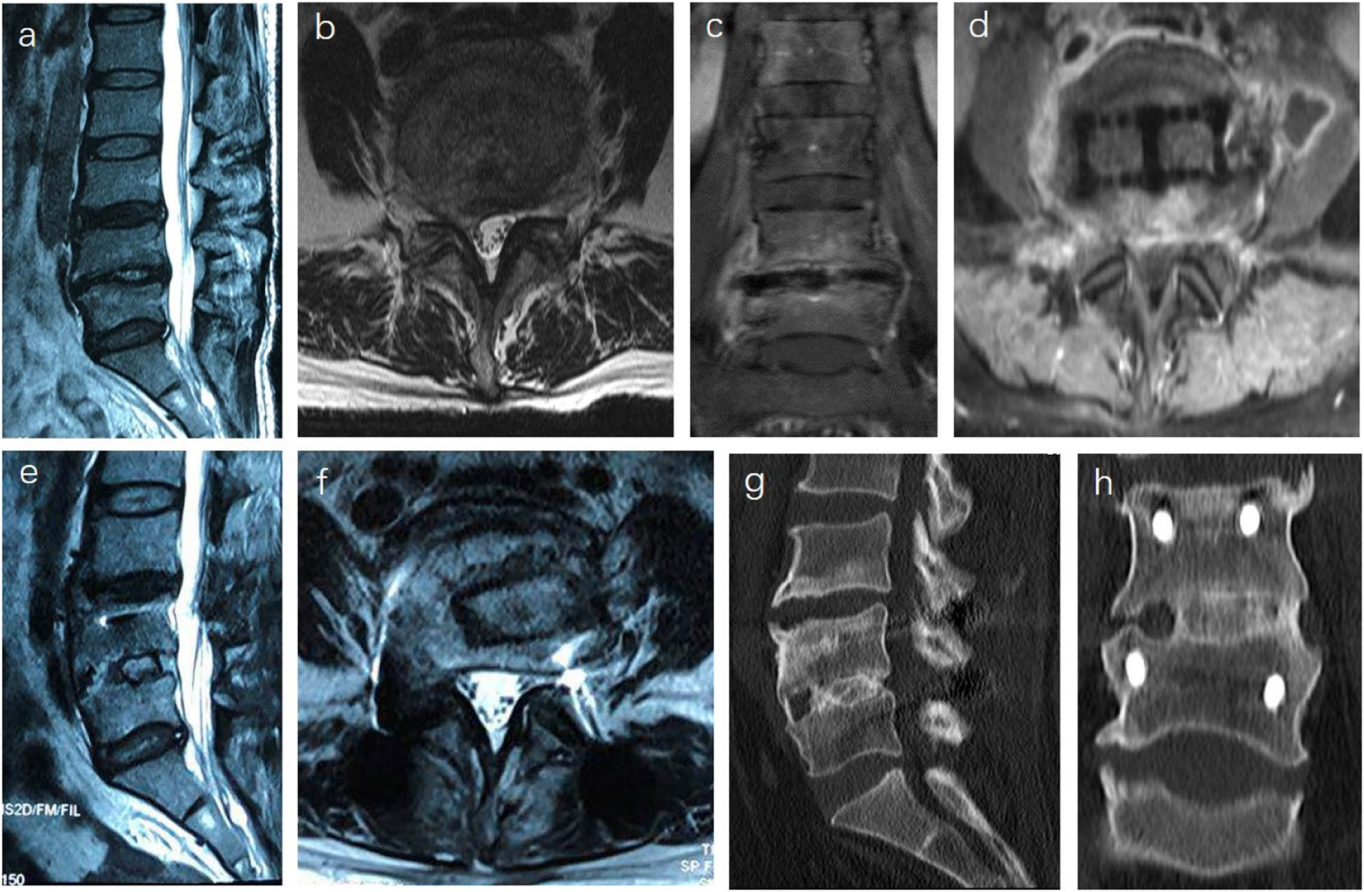
A case of intervertebral space infection. **a**, **b** Preoperative sagittal and axial MRI. **c**, **d** Coronal and axial MRI showed infection in L45 and intervertebral space collapse. **e**, **f** Sagittal and axial MRI after debridement and posterior internal fixation. **g**, **h** Sagittal and axial CT at one year after operation showed that bony fusion had been achieved

The probability of infection in LLIF is only 0.6–0.73% [25, 26]. The reasons for the lower incidence of infection may be related to less trauma, shorter operation time and passage operation. Infection usually occurs in patients with obesity or diabetes, and wound infection can be cured by conservative treatment. However, intervertebral space infection is rarely reported. Intervertebral space infection can be transmitted from the blood or gastrointestinal tract or directly. In order to prevent the infection from spreading to the posterior column of the spine, debridement through the original surgical incision is a better choice. In the early stage of infection, implants can be considered to be preserved. When the focus of infection is large and the infection is difficult to control, focal debridement, bone grafting and internal fixation are needed to maintain the stability of the spine and control the infection. Figure 2 shows a special case of spinal infection, and there is no obvious abnormality in hemogram and lumbar MRI before operation (Fig. 2a-b). Fever occurred after one-stage operation. Brucella agglutination test showed negative, lumbar nuclear magnetic resonance showed infection (Fig. 2c-d). After revision through the original surgical incision, pathology showed brucellosis infection.

### Vascular injury

The incidence of major vascular injuries occurring for LLIF performed in a larger case series involving a total of 4,607 patients is 0.4% [26]. Although its incidence is not high, it is very dangerous. If the space between psoas major muscle and vascular sheath is very narrow, vascular injury is easy to occur during OLIF. Once suspected vascular injury occurs during operation, it is necessary to stop bleeding thoroughly, otherwise it is easy to cause delayed bleeding after operation. The key to revision lies in the early detection that once the wound is painful and the blood pressure drops rapidly, timely and effective measures should be taken to stop the bleeding.

### Cage migration

**Fig. 3:**
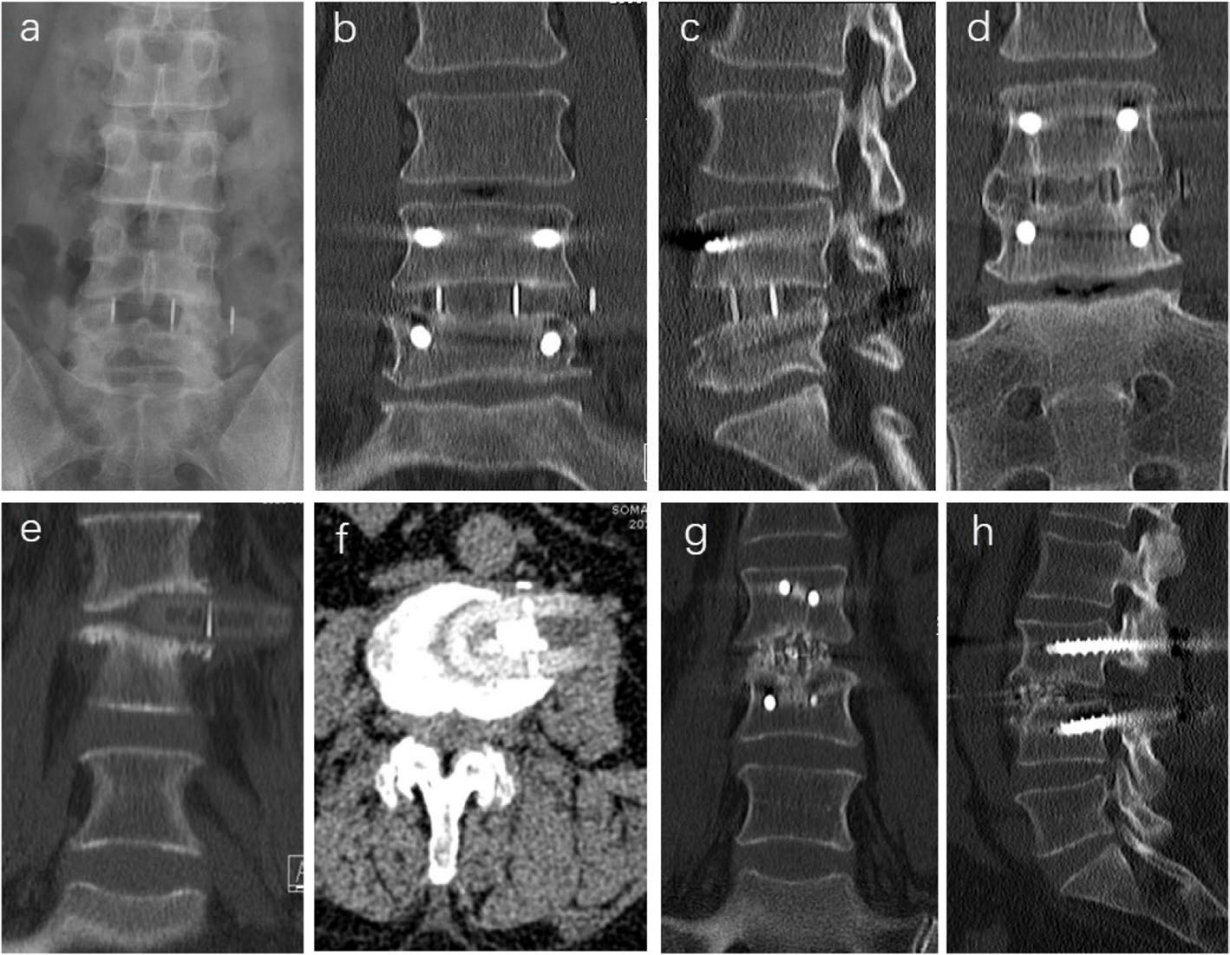
A case of cage displacement after LLIF **a**-**d**. **a** Cage displacement can be seen in anterior X-ray. **b**, **c** Coronal and sagittal CT of lumbar spine after revision. **d** Coronal CTshowed bony fusion at two years after operation. A case of cage prolapse after LLIF **e**-**h**. **e**, **f** Coronal and axial CT showed cage prolapse at three months after OLIF. **g**, **h** Coronal and sagittal CT after revision

In our study, the revision rate of patients with cage migration accounted for 1.44%. The causes of cage migration are mainly due to obesity, getting out of bed too early, small interbody fusion cage, abnormal vertebral body shape, improper bone graft material, osteoporosis, bony endplate injury and insufficient opening of contralateral annulus [27–29]. Cage migration usually occurs after SA-LLIF. Early stage of cage migration, because of small displacement and mild symptoms, the cage can be fixed by adding posterior internal fixation alone (Fig. 3a-d). If cage prolapse occurs, the prolapsed cage will oppress the lumbar plexus, and the protruded intervertebral space will collapse and cause lower limb symptoms. So, it is necessary to adjust the position of the cage from the original way and then apply internal fixation (Fig. 3e-h).

### Unsatistactory implant placement

**Fig. 4:**
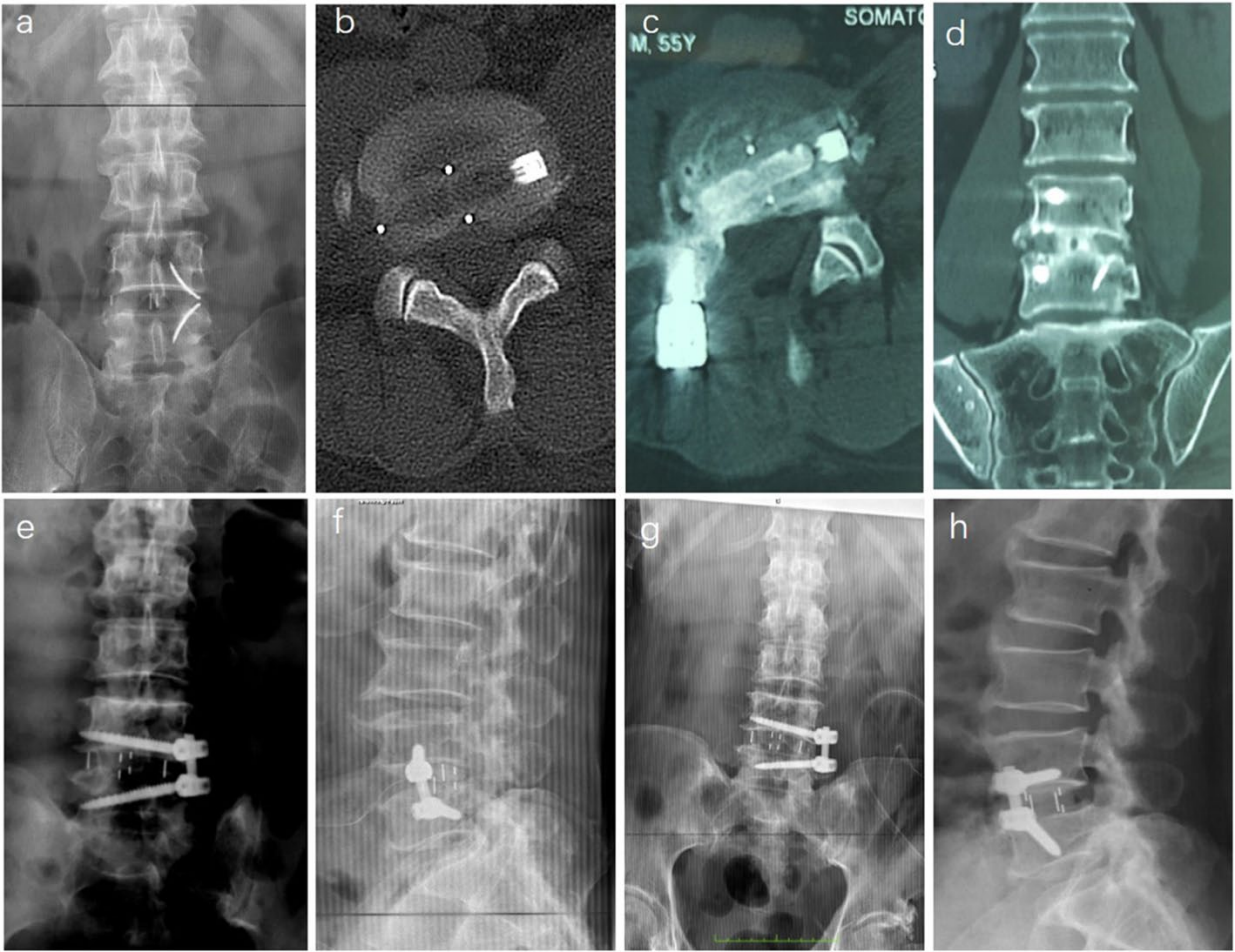
A case of right nerve root compression using TLIF revision **a**-**d**. **a** Anterior X-ray after XLIF. **b** Axial CT showed that the right nerve root was compressed by cage. **c**, **d** Axial and coronal CT showed direct decompression of nerves and fusion one year after right TLIF. A case of revision in which the position of the screw was adjusted through the original incision **e**-**h**. **e**, **f** Anterior and lateral X-ray after XLIF. **g**, **h** Anterior and lateral X-ray showed the position of screw was adjusted through the original incision, and the symptoms were relieved

Unsatistactory implant placement cases accounted for 14.3% of all revision cases. There are many reasons for unsatistactory implant placement, such as high iliac crest, psoas major hypertrophy, the establishment of lumbar bridge and other factors, it is difficult to straighten the channel, so the cage and inferior screws are not completely vertical. For the cases with persistent neurological symptoms, good results can be obtained by changing the position of implant placement and relieving nerve compression by two-stage operation. However, in some cases, due to the use of cage with fin device, it is difficult to remove it during revision, so we adopted posterior decompression or symptomatic side transforaminal lumbar interbody fusion (TLIF), which relieved the compression under direct vision and avoided the removal of cage. (Fig. 4)

### Internal spinal instrumentation displacement

**Fig. 5:**
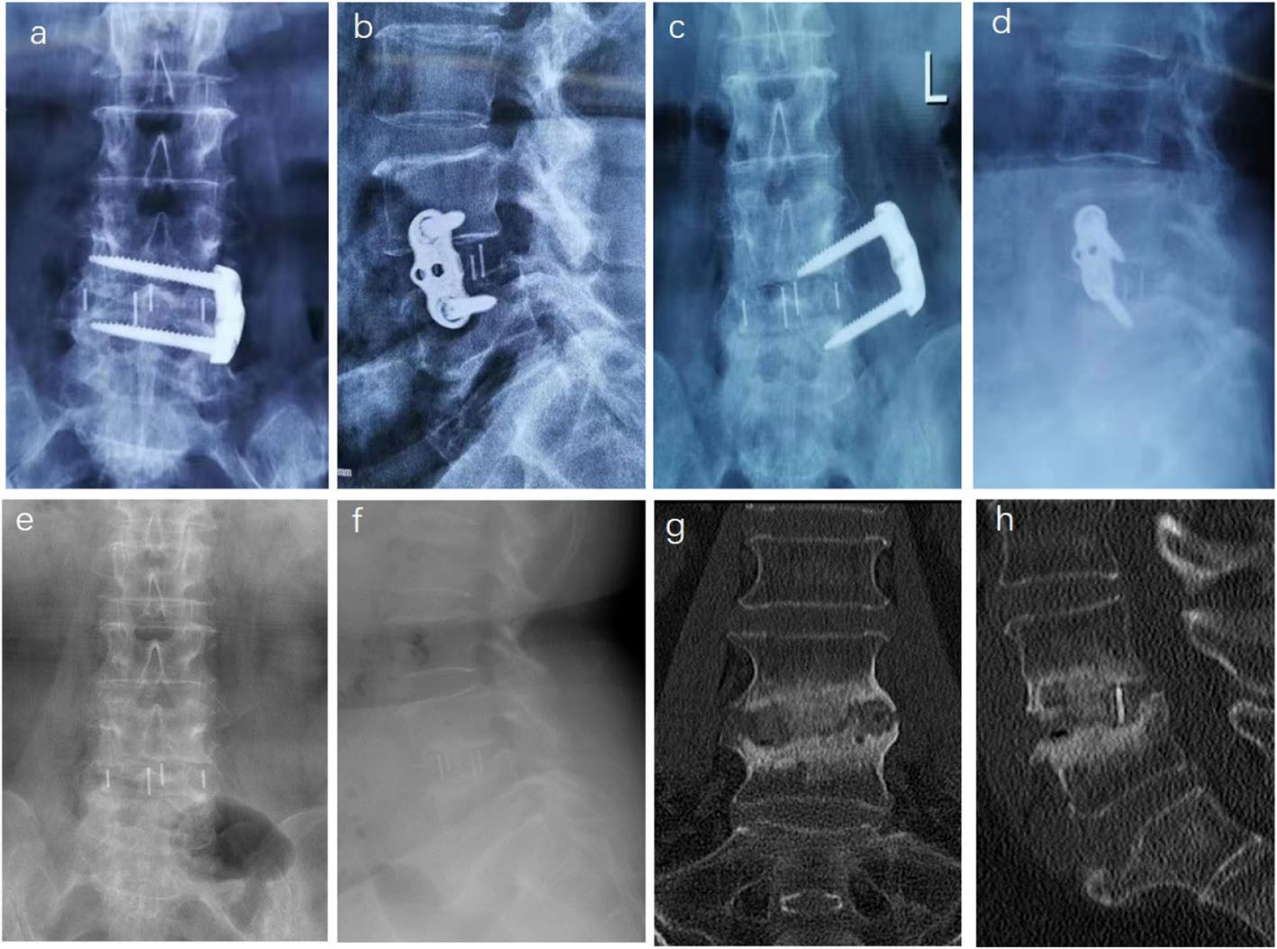
A case of failure of internal fixation. **a**, **b** Anterior and lateral X-ray after XLIF. **c**, **d** Anterior and lateral X-ray one month after operation. **e**, **f** Anterior and lateral X-ray after revision. **g**, **h** Coronal and sagittal CT one year after revision

The incidence of internal fixation failure and revision after LLIF is about 0.96%. The failure of spinal internal fixation is often caused by improper selection of operation or erroneous installation. If the posterior internal fixation is loose or broken, it is naturally necessary to replace the internal fixation after being removed from the original posterior incision. However, the direction of stress and the curvature of the lumbar spine should also be taken into account during revision to prevent recurrence. When lateral internal fixation is selected for one-stage operation, in addition to removing the original internal fixation, it is also necessary to consider increasing posterior pedicle screw fixation to maintain spinal stability. Figure 5 shows a case of lateral nailboard loosening, due to more underlying diseases and no obvious lower limb symptoms, we did not add internal fixation and advised him to increase bed rest time. During the postoperative follow-up, although there was a small amount of endplate collapse (Fig. 5f), the operative segment tended to be stable one year after operation, and there were no obvious lower limb symptoms (Fig. 5 g). Therefore, it is necessary to make a clear diagnosis before operation and fully estimate the stress that the implant can bear in the body; master the installation mode and application purpose of each component of the implant. For those patients with severe osteoporosis, posterior internal fixation should be chosen to provide stronger support, and strengthen the treatment of anti-osteoporosis.

Although there are studies have suggested that LLIF can be applied to some patients with mild to moderate spinal canal stenosis [11]. However, for severe spinal canal stenosis, some scholars believe that it is a taboo of LLIF [30, 31], and severe spinal canal stenosis is a risk factor for secondary decompression after LLIF [32]. In our study, there was a difference in the proportion of preoperative spinal canal stenosis between the revision group and the non-revision group, but this was not an independent risk factor for revision. Similar to the study of Hiyama et al. [33], we believe that preoperative severe spinal stenosis is not an absolute contraindication of LLIF, but LLIF may not be suitable for severe bony stenosis.

By reviewing the data of all patients undergoing revision after LLIF, we found that postoperative foraminal stenosis was an independent risk factor for revision. It is also one of the factors affecting the revision decision of the operator. According to the study of Wang et al. [34], LLIF can not decompress the nerve roots of patients with severe intervertebral foramen stenosis before operation. In addition to bony stenosis, we believe that endplate collapse is one factor leading to intervertebral foramen restenosis. Since LLIF completely increases the intervertebral height by inserting large cage, once the graft subsidences or moves, it will lead to the loss of intervertebral height and foraminal stenosis again, resulting in lower limb symptoms [28, 35, 36]. Displacement, loosening and fracture of the internal fixation will also lead to the weakening of the stabilization effect, causing foraminal stenosis and low back pain. In addition, excessive test model and improper choice of internal fixation are also factors leading to postoperative foraminal stenosis, which need further study in the future.

For the surgical efficacy of LLIF, health-related quality-of-life measurements decreased in varying degrees after operation. Timely revision operation can achieve satisfactory results, but the rehabilitation time of the revision operation is longer. Due to the small sample size and follow-up period, our research has multiple limitations. First of all, the factors that we can explore in multivariate analysis are limited. In addition, due to the lack of follow-up data for some patients, they were not included in this study, even if they may affect the results. In the statistics of complications, because of the short follow-up time, we did not summarize or discuss the long-term complications such as adjacent segmental degeneration. In the future, we should expand the sample size, prolong the follow-up period, and explore the long-term efficacy of LLIF.

## Conclusion

This study attempts to summarize the strategy of revision after lateral lumbar interbody fusion and test the curative effect. Lateral lumbar interbody fusion is a surgical method with less trauma, less blood loss and quick recovery after operation, but its indications need to be strictly observed and appropriate cases should be selected. Postoperative foraminal stenosis is a factor affecting the decision-making of postoperative revision. All kinds of postoperative complications should be dealt with according to different revision strategies in time, and satisfactory results can be obtained after operation.

## Data Availability

The datasets used and/or analysed during the current study are available from the corresponding author on reasonable request.

## Abbreviations

LLIF: Lateral lumbar interbody fusion
CT: Computed tomography
MRI: Magnetic resonance imaging
BMI: Body mass index
ODI: Oswestry disability index
VAS: Visual analogue scale
IDF: Indirect decompression failure
UBE: Unlateral biportal endoscoic
OLIF: Oblique lumbar interbody fusion
TLIF: Transforaminal lumbar interbody fusion
